# Transfection of the mutant *MYH9 *cDNA reproduces the most typical cellular phenotype of *MYH9*-related disease in different cell lines

**DOI:** 10.1186/1755-8417-1-5

**Published:** 2008-12-01

**Authors:** Emanuele Panza, Monica Marini, Alessandro Pecci, Francesca Giacopelli, Valeria Bozzi, Marco Seri, Carlo Balduini, Roberto Ravazzolo

**Affiliations:** 1 Medical Genetics Unit, Department of Gynecology, Obstetrics and Pediatrics, University of Bologna, Bologna, Italy; 2 Laboratory of Molecular Genetics, G. Gaslini Institute, Genova, Italy; 3 Department of Internal Medicine, IRCCS Policlinico S. Matteo, University of Pavia, Pavia, Italy; 4 Department of Pediatrics and CEBR, University of Genova, Genova, Italy

## Abstract

**Background:**

Heterozygous mutations of *MYH9*, encoding the Non-Muscular Myosin Heavy Chain-IIA (NMMHC-IIA), cause a complex disorder named *MYH9*-related disease, characterized by a combination of different phenotypic features. At birth, patients present platelet macrocytosis, thrombocytopenia and leukocyte inclusions containing NMMHC-IIA. Moreover, later in life some of them develop the additional features of sensorineural hearing loss, cataracts and/or glomerulonephritis that sometimes leads to end stage renal failure.

**Results:**

To clarify the mechanism by which the mutant NMMHC-IIA could cause phenotypic anomalies at the cellular level, we examined the effect of transfection of the full-length mutated D1424H *MYH9 *cDNAs. We have observed, by confocal microscopy, abnormal distribution of the protein and formation of rod-like aggregates reminiscent of the leukocyte inclusions found in patients. Co-transfection of differently labeled wild-type and mutant full-length cDNAs showed the simultaneous presence of both forms of the protein in the intracellular aggregates.

**Conclusion:**

These findings suggest that the NMMHC-IIA mutated in position 1424 is able to interact with the WT form in living cells, despite part of the mutant protein precipitates in non-functional aggregates. Transfection of the entire WT or mutant *MYH9 *in cell lines represents a powerful experimental model to investigate consequences of *MYH9 *mutations.

## Background

Heterozygous mutations in the *MYH9 *gene, encoding the Non-Muscle Myosin Heavy Chain IIA (NMMHC-IIA), are responsible for the recently defined *MYH9*-related disease (*MYH9*-RD) [1]. This entity includes clinical phenotypes previously classified as distinct disorders: May-Hegglin anomaly, Sebastian syndrome, Fechtner syndrome, and Epstein syndrome. All patients present since birth thrombocytopenia and leukocyte inclusions consisting of aggregates of NMMHC-IIA. However, during infancy or adult life many subjects develop the additional features of sensorineural hearing loss, cataracts, and/or progressive nephropathy leading to renal failure [1].

NMMHC-IIA is a conventional, non-sarcomeric myosin expressed in most cells and tissues, where it is involved in several functions including cytokinesis, cell motility, and maintenance of cell shape [2]. The N-terminal portion of NMMHC-IIA forms the myosin globular head, responsible for ATPase and actin-binding activity, while the C-terminal tail region regulates both dimerization of heavy chains in coiled-coil structures and association of myosin molecules into functional filaments [2].

The mechanisms by which *MYH9 *mutations cause *MYH9*-RD are poorly defined, and both haploinsufficiency and a dominant-negative effect of the mutated protein have been hypothesized [3-6]. In particular, studies on NMMHC-IIA tail fragments demonstrated that differently mutated proteins could interact to a different extent with WT counterparts to exert a dominant-negative biochemical effect [5]. Nevertheless, investigations on megakaryocytes and platelets from patients with *MYH9*-RD suggested that mutant NMMHC-IIA proteins could not be expressed in living cells, possibly because of their high instability [3,6]. Difficulties in addressing these issues include the unavailability of the entire NMMHC-IIA [7], so that experimental approaches were so far limited to *in vitro *studies on either N-terminal or C-terminal portions of the molecule [5].

Here we present the results of transfection with the entire NMMHC-IIA molecule both in wild-type (WT) and mutated form (D1424H) in COS-7 and HeLa cell lines.

## Methods

The full-length 5883 bp *MYH9 *cDNA was cloned using the Gateway system (Invitrogen). RNA was extracted from a lymphoblastoid cell line from a control individual. Five hundred nanograms of total RNA were specifically retrotranscribed in order to obtain *MYH9 *cDNA (Transcriptor First Strand cDNA Synthesis Kit, Roche). The resulting cDNA was used for PCR amplification. Primers, which included the attB1 and attB2 recombination sites, were:

Forward-AttB1: GGGGACAAGTTTGTACAAAAAAGCAGGCTCCATGGCACAGCAAGCTGCC;

Reverse-AttB2: GGGGACCACTTTGTACAAGAAAGCTGGGTCTTATTCGGCAGGTTTGGCC.

Forty nanograms of cDNA were used to amplify the entire *MYH9 *coding sequence.

The PCR fragment was introduced into plasmid vector pDONOR207 (Invitrogen) via the BP recombination reaction, according to the recommendations of the manufacturer, to generate the entry clone. Control of the resulting cDNA was performed by complete sequencing. Comparison of the sequence in our clone with the cDNA reported in the NCBI database (NM_002473) did not reveal any difference.

Site directed mutagenesis was performed using a commercial kit (Quick Change, Stratagene) to introduce a G to C transversion in position 4270, in order to obtain the D1424H mutant. WT and D1424H constructs were then used to perform LR recombination reactions with the Gateway system compatible expression vector pcDNA3.1/nV5-DEST (Invitrogen) and Gateway converted Flag vector to express N-terminally tagged V5, or Flag fusion proteins, respectively. The integrity of all constructs was confirmed by direct complete DNA sequencing.

COS-7 and HeLa cells were maintained in Dulbecco's modified Eagle's medium supplemented with 10% (v/v) fetal bovine serum (GibcoBRL), 2 mM L-glutamine, 100 U/ml penicillin, 100 μg/ml streptomycin at 37°C in a humidified atmosphere with 5% CO2. Transfections of both cell lines were performed using the Polyfect reagent (Quiagen), according to the manufacturer's instructions, on cells plated at 50–60% confluence and transfected at an estimated 80–90% confluence after 24 h. Cells were harvested 24, 48 and 72 hours after transfection and cytospun onto slides for immunofluorescence staining.

Standard peripheral blood smears were prepared from two patients with *MYH9*-RD caused by D1424H substitution of NMMHC-IIA. Both patients have been already reported [1]. The institutional review board of Fondazione IRCCS Policlinico San Matteo, University of Pavia, Italy, approved the study and patients gave written informed consent to the study.

The following primary antibodies (Abs) were used: rabbit polyclonal PR-B440P (Covance Research Products Berkeley, CA) recognizing the 12-residues sequence (GKADGAEAKPAE) of the C-terminus of NMMHC-IIA [8], mouse monoclonal NMG2 to a specific epitope of the N-terminus of NMMHC-IIA (a kind gift of Dr Saverio Sartore, Biomedical Sciences, University of Padua, Italy) [9]; rabbit polyclonal anti-flag (Sigma, St. Louis, MI), and mouse monoclonal anti-V5 (Invitrogen).

Secondary antibodies were goat anti-mouse or anti-rabbit conjugated with either Alexa Fluor 488 or Alexa Fluor 594 (Invitrogen). Confocal microscopy was performed through the TCS SPII confocal laser scanning microscopy system (Leica, Heidelberg, Germany), equipped with a Leica DM IRBE inverted microscope, as previously described [6]. Confocal optical sections were performed every 500 nm. Conventional fluorescence microscopy was performed through an Axioscope 2 Plus microscope (Carl Zeiss, Gottingen, Germany), as described [6].

## Results and discussion

The entire 5883 bp *MYH9 *cDNAs, both WT and D1424H form, have been transfected in COS-7 or in HeLa cell lines. Immunofluorescence staining showed that COS-7 cells, which constitutively do not express MYH9, expressed the transfected WT or D1424H NMMHC-IIAs, and that the signal of these molecules was stable for at least 72 hours after transfection. No NMMHC-IIA signal has been observed in non-transfected cells or in cells transfected with the vector alone. Transfected COS-7 cells were similarly labeled by antibodies specific either to the N-terminus or to the C-terminus of the protein, demonstrating that the entire WT or mutant NMMHC-IIA were expressed.

**Figure 1:**
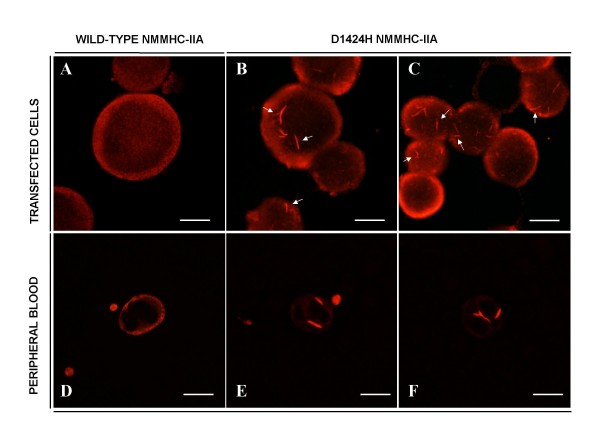
**Transfection of D1424H *MYH9 *in COS-7 or HeLa cells results in the formation of NMMHC-IIA aggregates similar to those of granulocytes of *MYH9*-RD patients carrying the same mutation**. A) COS-7 cells transfected with WT *MYH9 *cDNA. B) COS-7 cells transfected with D1424H *MYH9 *cDNA. C) HeLa cells transfected with D1424H *MYH9 *cDNA. D) Staining of granulocyte from an unaffected individual. E) and F) Staining of granulocytes from patients with the D1424H substitution obtained by labeling the peripheral blood smears for NMMHC-IIA. Scale bars correspond to 10 μm. Arrows indicate rod-like aggregates.

Confocal microscopy demonstrated that the signal of the respective protein tags (V5 for WT, flag for D1424H) was completely superimposable to the NMMHC-IIA signal, confirming that staining for the tags was reliable to assess both expression and intracellular distribution of the respective exogenous NMMHC-IIAs (data not shown). While in COS-7 transfected with WT *MYH9 *the NMMHC-IIA signal was uniformly distributed in the cytoplasm, in cells transfected with the D1424H construct NMMHC-IIA formed rod-like protein aggregates similar to those observable in granulocytes of patients carrying the D1424H substitution (Figure 1A and 1B). To show such similarity, peripheral blood smears were prepared from two patients with *MYH9*-RD caused by the D1424H substitution of NMMHC-IIA, compared with an unaffected individual (Figure 1D–F). Both patients had already been reported [1].

These aggregates of mutant NMMHC-IIA were present in most transfected cells, and they were usually evident over a diffuse cytoplasmic staining. When we transfected with the same constructs HeLa cells, which constitutively express NMMHC-IIA, we obtained the same results as in COS-7 (Figure 1C).

Thus, transfection of the entire *MYH9 *mutant cDNA reproduced in two different cell lines, independently from their endogenous myosin expression, the NMMHC-IIA aggregates that are typical of granulocytic cells in *MYH9*-RD [1,4,10]. Interestingly, a similar distribution of NMMHC-IIA was observed in tubular epithelia cells from the only patient with *MYH9*-RD whose renal biopsy was investigated by immunohystochemistry [10], suggesting that this behavior of mutant NMMHC-IIA could be common to different cell types *in vivo*.

**Figure 2:**
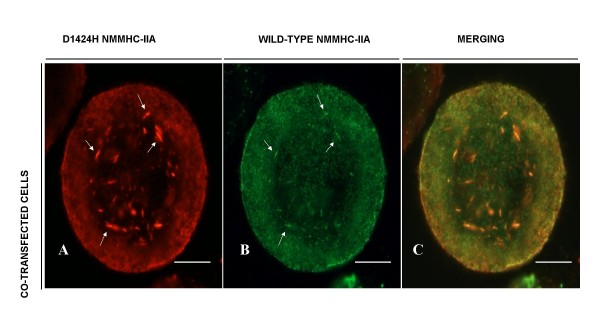
**Exogenous D1424H and WT NMMHC-IIA co-localize in co-transfected COS-7 cells**. A) COS-7 cells co-transfected with both D1424H and WT *MYH9 *cDNAs tagged at the N-terminal end with flag and V5 epitopes, respectively: red staining for the flag epitope (mutant). B) Same co-transfected cells for the V5 epitope (WT, green). C) Merging of images resulting from overlapping of the two channels. Scale bars correspond to 10 μm. Arrows indicate rod-like aggregates.

Since in cells of *MYH9*-RD patients both a WT and a mutant allele are present, we co-transfected COS-7 or HeLa cells with both WT and D1424H cDNAs and investigated intracellular localization of the two exogenous NMMHC-IIAs by double-labeling cells for the respective protein tags. In co-transfected cells the mutant NMMHC-IIA showed a distribution similar to that observed in cells transfected with mutant *MYH9 *alone, with part of the flag signal diffused in the cytoplasm and part organized in rod-like aggregates (Figure 2A). We noticed that aggregates were less frequent than in cells transfected with D1424H cDNA alone. Confocal analysis demonstrated that the V5 (WT) signal presented a high degree of co-localization with the flag (mutant) signal diffused in the cytoplasm, thus suggesting that transfected WT and D1424H molecules interacted in these cells (Figure 2A–C). Interestingly, the V5 signal was detected also at the level of some of the aggregates disclosed by the flag reaction, although it was less intense than the mutant signal (Figure 2A–C). This finding indicates that rod-like aggregates were mainly constituted by D1424H NMMHC-IIA, although WT protein was trapped into some of these structures. This observation is consistent with results previously obtained in granulocytes of patients with nonsense or frameshift *MYH9 *mutations resulting in a truncated NMMHC-IIA [4,6,11].

Results of cotransfection similar to those in COS-7 cells were also obtained in HeLa cells (not shown).

## Conclusion

These findings suggest that the NMMHC-IIA mutated in position 1424 is able to interact with the WT form in living cells, despite part of the mutant protein precipitates in non-functional aggregates. This observation is consistent with previous studies on an *in vitro *model, which demonstrated that the D1424N C-terminal fragment of NMMHC-IIA, when mixed with its WT counterpart, is able to form paracrystalline assemblies similar to those of the WT/WT interaction [5]. The fact that NMMHC-IIA mutated in position 1424 retains the ability to interact with WT protein represents the basis for it to exert a dominant-negative biochemical effect on the normal protein.

Transfection of the entire WT or mutant *MYH9 *in cell lines represents a promising experimental model to investigate consequences of *MYH9 *mutations in cells.

## Competing interests

The authors declare that they have no competing interests.

## Authors' contributions

RR proposed and supervised the study, analyzed data, and co-wrote the article; EP prepared the full-length cDNA construct and performed site specific mutagenesis; MM carried out transfection experiments; AP performed confocal microscopy analysis; FG collaborated in transfection experiments; VB collaborated in confocal microscopy analysis; MS supervised construct preparation, provided intellectual expertise, and co-wrote the article; CB supervised morphological study, provided intellectual expertise, and co-wrote the article. EP, MM and AP contributed equally to the work.
